# Effects of SARS-CoV-2 Variants on Maternal Infection and Severity: A Single-Center Experience

**DOI:** 10.7759/cureus.24201

**Published:** 2022-04-17

**Authors:** Behiye Deniz Kosovali, Busra Tezcan, Nevzat Mehmet Mutlu

**Affiliations:** 1 Department of Critical Care Medicine, Health of Science Ankara City Hospital, Ankara, TUR

**Keywords:** icu mortality, delta variant, intensive care unit, pregnant females, covid-19

## Abstract

Background and aims

Pregnant women are one of the vulnerable groups affected by COVID-19. With the mutation of the virus, the severity of the disease in this vulnerable group may vary in different waves of COVID-19 subtypes. The aim of this study is to define the demographic, clinical, laboratory, and mortality results of pregnant COVID-19 patients according to three time frames (March to December 2020, January to June 2021, and July to November 2021).

Materials and methods

The data of patients admitted to the ICU between March 23, 2020, and November 30, 2021, were retrospectively scanned. Pregnant patients with SARS-CoV-2 PCR test positivity or pregnant patients with COVID-19 who have a negative PCR test but symptoms of COVID-19 and radiological findings consistent with COVID-19 on thorax CT who need intensive care were included in the study. The patients were divided into three groups according to the dates when the Ministry of Health of the Republic of Turkey reported the variants of COVID-19 in Turkey. The nonvariant type was dominant in the first period (March to December 2020), alpha and beta variants were dominant in the second period (January to June 2021), and the delta variant appeared in the last period (July to November 2021). Demographic, clinical, and laboratory findings at the first admission to the ICU and mortality rates of the patients were recorded.

Results

PCR test was performed in all 109 patients, of whom 101 were PCR test positive. In other eight patients, despite the negative PCR test, thorax CT findings were typical of COVID-19 pneumonia, and other bacterial and viral agents were also excluded. The mean age of the patients was 30.53 years, the mean APACHE II score was 9.68, and the mean gestational age was 28.55 weeks. Around 72.5% of the patients were in the third trimester. Of the 101 PCR-positive patients, 20.2% were delta variants, 16.5% alpha or beta variants, and 63.3% were of unknown variants. Five of the patients were vaccinated. The most common symptom was dyspnea (94.5%), and the most common comorbidity was hypothyroidism (9.17%). Invasive mechanical ventilation (IMV) was needed in 44.95% of pregnant patients. The distribution of pregnant patients admitted to the ICU according to the periods March to December 2020, January to June 2021, and July to November 2021 was 16.5%, 21.1%, and 62.4%, respectively (p<0.001). Two groups of patients were compared: those that survived versus those that deceased. Variables predicting mortality were APACHE score, IMV requirement, length of stay in the ICU, prone positioning, Anakinra treatment, and ECMO (extracorporeal membrane oxygenator) requirement, which were significantly higher in the deceased group than in the living group (p<0.001, p<0.001, p=0.001, p<0.001, p<0.001, and p=0.001, respectively). There was no significant difference between the patients' age, gestational age, variants, treatments other than Anakinra, and the number of patients admitted to the ICU in the three periods (p=0.667, p=0.174, p=0.904, and p=0.605, respectively).In the multiple logistic regression analysis for mortality, high APACHE II score and IMV requirement were found as risk factors for mortality.

Conclusion

In the last period of delta variant predominance, pregnant COVID-19 patients were admitted to the ICU significantly more frequently than in the first two periods. Mechanical ventilation requirement and high APACHE II score were determined as risk factors for mortality.

## Introduction

Coronavirus infectious disease 2019 (COVID-19), caused by severe acute respiratory syndrome coronavirus 2 (SARS-CoV-2), can create different symptoms, ranging from flu-like symptoms to respiratory failure. The clinical course may be asymptomatic, mild, severe, or multiorgan dysfunction syndrome with COVID-19 in intensive care unit (ICU) [1]. Since the beginning of the pandemic, many children, adults, young, and geriatric people with or without comorbidities have been infected with SARS-CoV-2. Normally, pregnant women are considered healthy individuals. However, in a process such as a pandemic, pregnant women have become one of the vulnerable groups in terms of COVID-19 due to the physiological changes that occur during pregnancy. In the COVID-19 pandemic that has been going on since December 2019, the virus mutated many times and formed different variants. According to the characteristics of these variants, the transmission rate and severity of the disease and the population it affected varied [2].

After March 11, 2020, when the first COVID-19 case was reported in Turkey, hygiene, mask, social distance, and intermittent lockdown rules were applied in order to prevent the increase in cases across the country [3]. With these precautions, the aim was to protect vulnerable groups such as the elderly, those with multiple comorbidities, and pregnant women from COVID-19. In the later periods of the pandemic, the lockdown was lifted in all age groups and the normalization period began (June 2021 onward). During the normalization period, vaccine program, which was developed against COVID-19, were added to mask, hygiene, and social distance measures (December 2021). Despite these measures, the waves in the number of COVID-19 cases and the emergence of variants of the virus followed an increase or decrease in the number of cases and the severity of the disease from time to time in Turkey as in the rest of the world. As COVID-19 continues, it has been reported that COVID-19 increases the risk of maternal and perinatal morbidity and that there is an increase in hospital and/or intensive care hospitalizations due to respiratory failure of pregnant women [4,5].

The aim of this study is to define the demographic, clinical, laboratory, and mortality outcomes of pregnant COVID-19 patients who were admitted to the ICUs of Ankara City Hospital, a tertiary pandemic center from March 23, 2020, when the pandemic started, until November 30, 2021, according to periods. The data were divided into three time periods according to the predominant COVID-19 strain: March to December 2020, January to June 2021, and July to November 2021.

## Materials and methods

**Figure 1:**
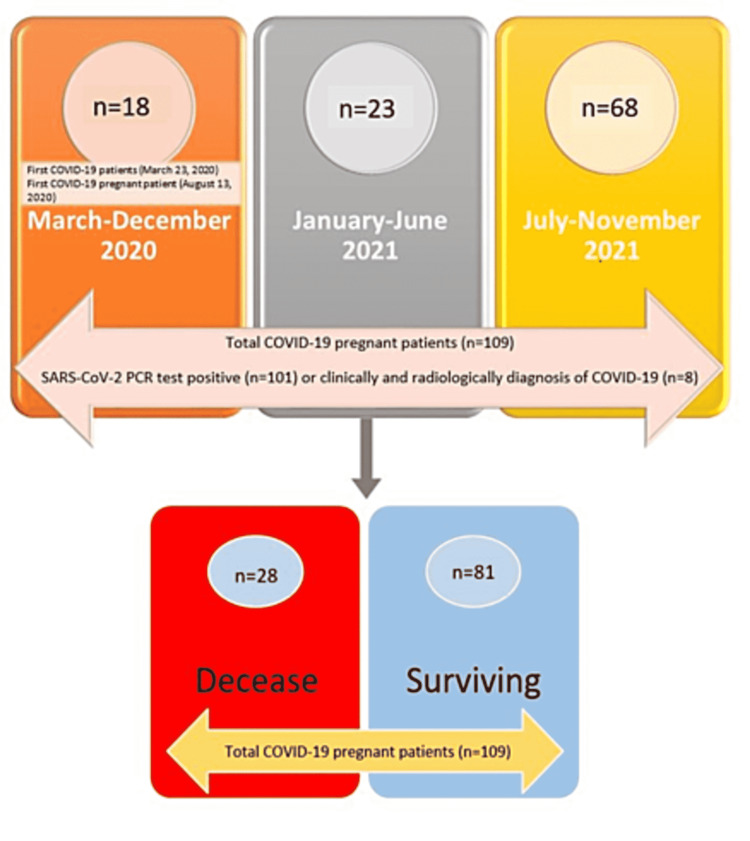
Inclusion criteria and compared groups

The data of the patients who were admitted to the ICU between March 23, 2020, and November 30, 2021, after obtaining the ethics committee approval (ethics committee no: E2-21-860), were retrospectively collected. Pregnant patients with SARS-CoV-2 polymerase chain reaction (PCR) test positivity or pregnant patients with COVID-19 who had a negative PCR test but symptoms of COVID-19 and radiological findings consistent with COVID-19 on chest X-ray or computed tomography (CT) of the thorax who need intensive care were included in the study (Figure 1).

The following data were recorded: patients' age, APACHE (Acute Physiology and Chronic Health Evaluation) II score in the first 24 hours in ICU, gestational age and trimester of pregnancy, singleton/multiple pregnancy, PCR test results, variant type (delta, others [alpha or beta], unknown [unchecked]), COVID-19 vaccination status (vaccinated with one or two doses of inactivated [Sinovac] or mRNA vaccines [Biontech], unvaccinated, unknown).

Further symptoms, comorbidities, need for invasive mechanical ventilation (IMV), length of stay in IMV, length of stay in ICU, supportive treatments in intensive care, nasal/mask oxygen, high-flow nasal cannula oxygen (HFO), noninvasive mechanical ventilation (NIMV), prone position, extracorporeal membrane oxygenator (ECMO), need for tracheotomy and hemodiafiltration (HDF), development of pneumothorax, termination of pregnancy, cesarean section time (before or after ICU admission), abortion, or whether pregnancy continued were also recorded.

Treatments for COVID-19 including antiviral agents (favipiravir, remdisevir, lopinavir/ritonavir), systemic steroid, interleukin-1 (IL-1) receptor antagonist (Anakinra), hydroxychloroquine, low molecular weight heparin (LMWH), colchicine, aspirin, other treatments, and mortality rates were recorded.

Laboratory data on admission to the ICU were recorded, including blood cell count, leukocytes, lymphocytes, neutrophils, hemoglobin, hematocrits, platelets, neutrophil/lymphocyte ratio, procalcitonin, C-reactive protein (CRP), interleukin-6 (IL-6), fibrinogen, ferritin, troponin, urea, creatinine, aspartate aminotransferase (AST), alanine aminotransferase (ALT), albumin, lactate dehydrogenase (LDH), and arterial blood gas analysis (pH, PaO_2_, PaCO_2_, bicarbonate, SpO_2_, lactate).

Patients admitted to the ICU as of March 23, 2020, were included in the study. However, the first COVID-19 pregnant woman was admitted to the ICU on August 13, 2020. The patients were divided into three groups according to the dates when the Ministry of Health of the Republic of Turkey reported the variants of COVID-19 in Turkey [6]. The nonvariant type was dominant in the first period (March 23 to December 30, 2020), alpha and beta variants were dominant in the second period (January 1 to June 30, 2021), and delta variant appeared in the last period (July 1 to November 30, 2021). Obtained results were compared between groups. All patients were followed up until ICU discharge or death.

Statistical analysis

Numerical data are summarized as mean ± standard deviation along with median (minimum-maximum), whereas frequency and percentage, n (%), were used for categorical data. Mann-Whitney U test or Kruskal-Wallis test was used for group comparisons regarding numerical variables. Dunn's test of multiple comparisons was used following a significant Kruskal-Wallis test. Groups were compared regarding a categorical variable using Fisher’s exact test or a chi-square test. Multiple logistic regression was performed to determine the risk factors of mortality. Backward stepwise selection algorithm was performed to determine the final model from potential risk factors found significant from univariate analysis. Odds ratio (OR) along with 95% confidence interval (CI), model fit (Hosmer-Lemeshow goodness of fit test result), and correct classification rate as model accuracy were reported. A p-value of <0.05 was considered as statistically significant. R statistical programming language (version 3.6.1, R Core Team, R Foundation for Statistical Computing, Vienna, Austria) was used for statistical analyses.

## Results

A total of 109 pregnant COVID-19 patients were included in the study; 101 pregnant women were diagnosed with PCR test positivity. Eight patients with negative PCR test but symptoms of COVID-19 (such as cough, sore throat, fever, dyspnea, etc.) and ground-glass opacification, consolidation, and crazy paving appearance on thorax CT, which are typical findings for COVID-19, and whose CT was reported as typical COVID-19 by radiologists, were also diagnosed with COIVID-19. The mean age of the patients was 30.53 years, the mean APACHE II score was 9.68, and the mean gestational age was 28.55 weeks. Two pregnant patients were in the first trimester of pregnancy. Therefore, data of first- and second-trimester patients were evaluated in the same group (27.5%). In addition, 72.5% of the patients were in the third trimester. Gestational trimesters of the pregnant patients admitted to the ICU between the three periods were similar (p=0.825). The admission rate of pregnant COVID-19 patients to the ICU by trimester and periods is shown in Table 1.

**Table 1:** Comparison of demographic and clinical characteristics of patients according to periods Data are represented as mean ± standard deviation or number (%). P-values are based on the Kruskal-Wallis test followed by Dunn’s multiple comparison test. ^a^Significantly different than August to December 2020^. ^bSignificantly different than January to June 202^1. ^¥Chi-square test APACHE, Acute Physiology and Chronic Health Evaluation; PCR, polymerase chain reaction; DM, diabetes mellitus; HT, hypertension; HFC, high-flow nasal cannula; IMV, invasive mechanical ventilation; ECMO, extracorporeal membrane oxygenation; HDF/CRRT, hemodiafiltration/continuous renal replacement therapy; C/S, cesarean section; ICU, intensive care unit; MV, mechanical ventilation; LMWH, low molecular weight heparin

Variables	Total	March to December 2020	January to June 2021	July to November 2021	p-Value
n (%)	109 (100)	18 (16.5)	23 (21.1)	68 (62.4)	<0.001
Age (mean±SD)	30.53±5.56	30.17±6.34	29.22±5.73	31.07±5.29	0.279
APACHE II (mean±SD)	9.68±9.01	11.94±11.85	8.52±7.06	9.47±8.78	0.718
Gestational age (mean±SD)	28.55±6.59	27.28±7.93	27.43±6.29	29.26±6.3	0.453
First and second trimesters	30 (27.5)	6 (33.3)	6 (26.1)	18 (26.5)	0.825
Third trimester	79 (72.5)	12 (66.7)	17 (73.9)	50 (73.5)	
Singleton pregnancy	107 (98.2)	17 (94.4)	23 (100)	67 (98.5)	0.347
Multiple pregnancy	2 (1.8)	1 (5.6)	0 (0)	1 (1.5)	
PCR test positive	101 (92.7)	15 (83.3)	21 (91.3)	65 (95.6)	0.162
Delta variant	22 (20.2)	0 (0)	0 (0)	22 (32.35)^a,b^	<0.001
Others (alpha or beta)	18 (16.5)	0 (0)	3 (13.0)	15 (22.1)^a,b^	
Unknown (variant unchecked)	69 (63.3)	18 (100)	20 (86.9)	31 (45.6)^a,b^	
Vaccinated	5 (4.6)	1 (5.6)	1 (4.3)	3 (4.4)	0.302
Unvaccinated	64 (58.7)	13 (72.2)	10 (43.5)	41 (60.3)	
Unknown	40 (36.7)	4 (22.2)	12 (52.2)	24 (35.3)	
Dyspnea	103 (94.5)	17 (94.4)	22 (95.6)	64 (94.1)	1.000
Cough	33 (30.3)	3 (16.7)	7 (30.4)	23 (33.8)	0.371^¥^
Fever	16 (14.7)	3 (16.7)	3 (13.0)	10 (14.7)	1.000
Myalgia/joint pain	4 (3.7)	0 (0)	1 (4.3)	3 (4.4)	1.000
Headache	3 (2.7)	0 (0)	0 (0)	3 (4.4)	0.750
Nausea/vomiting	5 (4.6)	1 (5.6)	1 (4.3)	3 (4.4)	1.000
Others	7 (6.4)	2 (11.1)	0 (0)	5 (7.3)	0.277
DM	2 (1.8)	0 (0)	1 (4.3)	1 (1.5)	1.000
HT	2 (1.8)	0 (0)	0 (0)	2 (2.9)	1.000
Hypothyroidism	10 (9.2)	1 (5.6)	4 (17.4)	5 (7.3)	0.314
HFC	86 (78.8)	14 (77.8)	19 (82.6)	53 (77.9)	0.892
IMV	49 (44.9)	9 (50)	7 (30.4)	33 (48.5)	0.287^¥^
Nasal/mask oxygen	22 (20.2)	4 (22.2)	5 (21.7)	13 (19.1)	0.890
ECMO	7 (6.4)	1 (5.6)	1 (4.3)	5 (7.3)	1.000
Tracheotomy	4 (3.7)	2 (11.1)	1 (4.3)	1 (1.5)	0.093
Prone position (IMV)	20 (18.3)	5 (27.8)	3 (13)	12 (17.6)	0.462
HDF/CRRT	3 (2.7)	0 (0)	1 (4.3)	2 (2.9)	1.000
Pneumothorax	10 (9.2)	2 (11.1)	4 (17.4)	4 (5.9)	0.195
C/S before ICU admission	47 (43.1)	9 (50)	8 (34.8)	30 (44.1)	0.618
C/S after ICU admission	31 (28.4)	3 (16.7)	6 (26.1)	22 (32.3)	
Pregnancy continued	27 (24.8)	5 (27.8)	8 (34.8)	14 (20.6)	
Spontaneous abortion	4 (3.7)	1 (5.6)	1 (4.3)	2 (2.9)	
Length of stay in ICU (day) (mean±SD)	10.42±9.45	11.67±10.51	12.43±12.06	9.41±8.08	0.759
Duration of MV (day) (mean±SD)	9.69±8.35	8.56±9.13	16.71±8.56	8.52±7.58	0.058
Live birth	70 (64.2)	11 (61.1)	13 (56.5)	46 (67.6)	0.849
Abortion	9 (8.3)	2 (11.1)	3 (13)	4 (5.88)	
Intrauterine exitus	7 (6.4)	1 (5.6)	1 (4.3)	5 (7.3)	
Pregnancy continued	23 (21.1)	4 (22.2)	6 (26.1)	13 (19.1)	
Antiviral	63 (57.8)	17 (94.4)	14 (60.9)	32 (47.06)^a,b^	0.001^¥^
Anakinra	37 (33.9)	6 (33.3)	6 (26.1)	25 (36.7)	0.645^¥^
Hydroxychloroquine	13 (11.9)	5 (27.8)	8 (34.8)	0 (0)	<0.001
Aspirin	44 (40.4)	5 (27.8)	6 (26.1)	33 (48.5)	0.081^¥^
LMWH	106 (97.2)	17 (94.4)	22 (95.6)	67 (98.5)	0.316
Colchicine	25 (22.9)	4 (22.2)	7 (30.4)	14 (20.5)	0.644
Other	12 (11)	4 (22.2)	3 (13)	5 (7.3)	0.163
Steroid	102 (11)	16 (88.9)	23 (100)	63 (92.6)	0.673
Maternal mortality	28 (25.6)	5 (27.8)	4 (17.4)	19 (27.9)	0.605

Of the patients, 107 were singleton pregnancies. Delta variant was detected in 20.2% of 101 patients with a positive PCR test, and other variants (alpha and beta) were detected in 16.5%. The variant type was not assessed in 63.3% of the patients. Five of the patients were vaccinated (one pregnant patient with two doses of inactive vaccine [Sinovac], two pregnant patients with a single dose of inactive vaccine [Sinovac], and two pregnant patients with one dose of mRNA vaccine [Biontech]; 64 of them were unvaccinated. Vaccination status of 40 patients could not be reached. The most common symptom in all patients was dyspnea (94.5%), and the most common comorbidity was hypothyroidism (9.17%). Other comorbidities were gestational diabetes (1.8%) and hypertension (1.8%). None of the COVID-19 pregnant patients had preeclampsia or eclampsia. IMV was required in 44.95% of the patients, prone position was applied in 18.35% of the patients after intubated, and ECMO was established in 6.42% of them.

Overall, 6.42% of the fetuses died due to intrauterine exitus, and abortion occurred in 8.26% of them. Among the antiviral agents, favipiravir, remdesivir, and lopinavir/ritonavir were given to 57.8% of the patients, Anakinra to 33.94%, LMWH to 97.25%, and systemic steroid for maternal survival in 92.7%. The distribution of pregnant patients admitted to the ICU according to the periods March to December 2020, January to June 2021, and July to November 2021 was 16.5%, 21.1%, and 62.4%, respectively (p<0.001). Table 2 shows the p-values of the patients admitted to the ICU when the periods were compared as the two groups.

**Table 2:** The p-values of multiple comparisons of the rate of patients admitted to the intensive care unit First period: March 23, 2020, to December 31, 2020; second period: July 1, 2021, to June 30, 2021, third period: July 1, 2021, to November 30, 2021

	p-Value
First vs second period	0.435
First vs third period	<0.001
second vs third period	<0.001

When the results of the patients in the three periods were compared, no difference was found in terms of demographic and clinical characteristics and mortality rates. However, there was a statistical difference between the periods determined in terms of variants, and the delta variant was detected only in the period of July to November 2021 (p<0.001).

While antiviral agents were minimally administered in the last period, hydroxychloroquine was not given to any patients during the last period (p=0.001 and <0.001, respectively). Other treatments given were as follows: cytokine filter (n=3), iloprost (n=2), dornase alfa (n=1), immune plasma (n=1), and intravenous immunoglobulin (n=9). LMWH was not given to a patient because of an epidural hematoma that occurred after an out-of-vehicle traffic accident. LMWH was not applied to the other two patients in the postoperative period, and these patients were discharged from the ICU on the first postoperative day. While there was no difference in mortality between the three periods, the overall mortality rate was 25.6% (p=0.605) (Table 1). One of the five vaccinated pregnant patients died. She was a patient in her third trimester of pregnancy with a single dose of inactive vaccine (Sinovac) and no additional comorbidities.

When the patients were compared as the deceased and surviving groups, the APACHE II score was found to be significantly higher in the deceased group (p<0.001). Length of stay in the ICU, IMV and ECMO requirement, post-intubation prone position, and tracheostomy applications were also significantly higher in the deceased group (p=0.001, p<0.001, p=0.001, p=0.004, p<0.001, and p=0.017, respectively) (Table 3).

**Table 3:** Comparison of demographic characteristics of deceased and surviving patients Data are represented as mean ± standard deviation or as number (%). ^¥^Chi-square test. APACHE, Acute Physiology and Chronic Health Evaluation; DM, diabetes mellitus; HT, hypertension; HFC, high-flow nasal cannula; IMV, invasive mechanical ventilation; ECMO, extracorporeal membrane oxygenation; HDF/CRRT, hemodiafiltration/continuous renal replacement therapy; C/S, cesarean section; ICU, intensive care unit; MV, mechanical ventilation; LMWH, low molecular weight heparin

Variables	Decease (n=28)	Surviving (n=81)	p-Value
Age (mean±SD)	30.36±5.80	30.59±5.52	0.667
Gestational age (mean±SD)	27.14±6.46	29.04±6.60	0.174
APACHE II (mean±SD)	18.14±12.18	6.75±5.09	<0.001
Delta	6 (21.43)	16 (19.75)	0.904
Others	5 (17.86)	13 (16.05)	
Unknown	17 (60.71)	52 (64.20)	
Vaccinated	1 (3.57)	4 (4.94)	0.485
Unvaccinated	14 (50)	50 (61.73)	
Unknown	13 (46.43)	27 (33.33)	
first and second trimesters	9 (32.14)	21 (25.93)	0.697^¥^
Third trimester	19 (67.86)	60 (74.07)	
C/S before ICU admission	10 (35.71)	37 (45.68)	<0.001
C/S after ICU admission	15 (53.57)	16 (19.75)	
Pregnancy continued	1 (3.57)	26 (32.10)	
Spontaneous abortion	2 (7.14)	2 (2.47)	
Length of stay in ICU (day)	16.25±11.46	8.41±7.76	0.001
Duration of MV (day)	11.23±9.59	7.96±6.44	0.482
HFC	24 (85.71)	62 (76.54)	0.449^¥^
IMV	26 (92.80)	23 (28.40)	<0.001^¥^
Nasal/mask oxygen	1 (3.57)	21 (25.93)	0.023^¥^
ECMO	6 (21.43)	1 (1.23)	0.001
Tracheotomy	4 (14.29)	0 (0)	0.004
Prone position (IMV)	12 (42.86)	8 (9.88)	<0.001^¥^
HDF/CRRT	2 (7.14)	1 (1.23)	0.161
Pneumothorax	6 (21.43)	4 (4.94)	0.017
Live birth	50 (61.73)	20 (71.43)	0.001
Abortion	4 (4.94)	5 (17.86)	
Intrauterine exitus	4 (4.94)	3 (10.71)	
Pregnancy continued	23 (28.4)	0 (0)	
Steroid	28 (100)	73 (90.12)	0.486
Antiviral	14 (50)	49 (60.49)	0.455^¥^
Anakinra	18 (64.29)	19 (23.46)	<0.001^¥^
Plaquanil	4 (14.29)	9 (11.11)	0.737
Ecoprin	16 (57.14)	28 (34.57)	0.061^¥^
DMAH	28 (100)	78 (96.30)	0.568
Colchicine	9 (32.14)	16 (19.75)	0.279^¥^
Other	6 (21.43)	6 (7.41)	0.073
ICU period	5 (17.86)	13 (16.05)	0.605
March to December 2020			
January to June 2021	4 (14.29)	19 (23.46)	
July to November 2021	19 (67.86)	49 (60.49)	

Of the given treatment agents, only Anakinra, which is an immunosuppressive and an IL-1 antagonist used during the cytokine storm period in COVID-19, was used significantly more frequently in the deceased group (p<0.001). When the laboratory values of the deceased and surviving groups were compared, the leukocyte, lymphocyte, and thrombocyte counts were significantly lower in the deceased group (p=0.036, p=0.010, p=0.024, respectively), while the procalcitonin, CRP, IL-6, ferritin, AST, and LDH values were significantly higher in this group (p=0.015, p=0.002, p<0.001, p=0.025, p=0.040, and p=0.020, respectively) (Table 4).

**Table 4:** Comparison of laboratory values of deceased and surviving groups Data are represented as mean ± standard deviation. Normal values of variables are indicated in parentheses. All variables are the first laboratory data of the patient on admission to the ICU. Hb, hemoglobin; Htc, hematocrit; Plt, platelet; N/L, neutrophil/lymphocyte ratio; CRP, C-reactive protein; IL-6, interleukin-6; AST, aspartate aminotransferase; ALT, alanine aminotransferase; LDH, lactate dehydrogenase; PaO_2_, partial pressure of oxygen; PaCO_2_, partial pressure of oxygen; HCO_3_, bicarbonate; SpO_2_, oxygen saturation

Variables	Total (n=109)	Decease (n=28)	Surviving (n=81)	p
Leukocytes x10^9^/L (3.6-10.5)	11.04±4.12	9.76±3.9	11.49±4.12	0.036
Lymphocyte, x10^9^/L (1.1-4)	0.85±0.42	0.69±0.38	0.9±0.42	0.010
Neutrophil, x10^9^/L (1.5-7.7)	9.62±3.82	8.58±3.62	9.98±3.85	0.073
Hb, g/dL (12.5-17.2)	10.84±1.38	11.16±1.35	10.73±1.38	0.182
Htc, % (37-49)	32.87±4.06	33.7±3.78	32.58±4.14	0.250
Plt, x10^9^/L (160-400)	283.8±102.53	248.71±108.08	295.93±98.33	0.024
N/L ratio	13.59±8.23	16.07±11.6	12.74±6.56	0.346
Procalcitonin, µg/L (< 0.16)	0.67±3.35	0.87±2.27	0.61±3.65	0.015
CRP, g/L (0-0.005)	0.09±0.06	0.12±0.05	0.08±0.05	0.002
IL-6, pg/mL (0-3.4)	97.11±526.78	269.74±1030.09	37.44±49.62	<0.001
Fibrinogen, g/L (1.7-4.2)	5±1.39	4.95±1.19	5.01±1.46	0.956
Ferritin, µg/L (22-322)	181.41±273.23	263.76±272.49	152.94±269.32	0.025
D-dimer, mg/L (<0.55)	3.76±6	4.5±7.35	3.51±5.49	0.243
Troponin, ng/L (<45)	34.25±127.9	81.43±224.13	17.94±63.9	0.297
Urea, mg/dL (19-49)	19.85±13.49	18.79±18.74	20.22±11.25	0.049
Creatinine, mg/dL (0.7-1.3)	0.49±0.26	0.48±0.09	0.49±0.3	0.363
AST, U/L (<35)	62.5±63.78	72.82±59.58	58.93±65.14	0.040
ALT, U/L (<50)	41.56±61.77	36.11±30.78	43.44±69.4	0.890
Albumin, g/L (32-48)	32±3.67	32.21±3.15	31.93±3.85	0.531
LDH, U/L (32-48)	433.06±142.63	497.04±185.69	410.95±117.9	0.020
pH (7.37-7.45)	7.42±0.07	7.4±0.07	7.42±0.07	0.232
PaO_2_, mmHg (70-100)	54.34±24.18	59.45±32.63	52.58±20.45	0.563
PaCO_2_, mmHg (35-46)	32.39±6.82	31.02±7.24	32.86±6.65	0.158
HCO_3_mmol/L (21-26)	20.44±3.65	18.94±3.97	20.95±3.41	0.075
SpO_2_, % (>96)	77.39±17.94	79.15±15.96	76.78±18.63	0.739
Lactate, mmol/L (<16)	1.83±0.77	1.83±0.79	1.82±0.77	0.986

In the multiple logistic regression analysis for mortality, one unit increase in APACHE II score and IMV requirement were determined as risk factors for mortality (Table 5).

**Table 5:** Multiple logistic regression for mortality *Age and pregnancy week adjusted results. Correct classification rate (accuracy) of the unadjusted model: 83.6%; Hosmer-Lemeshow goodness of fit χ2 = 6.28; SD=8; p=0.616 (model fits the data). Correct classification rate (accuracy) of the age and pregnancy week adjusted model: 83.6%; Hosmer-Lemeshow goodness of fit χ2 = 12.77; SD=8; p=0.120 (model fits the data). OR, odds ratio; CI, confidence interval; APACHE, Acute Physiology and Chronic Health Evaluation; IMV, invasive mechanical ventilation

	OR (95%CI)	p-Value	OR (95%CI)*	p-Value*
APACHE II score (1 unit increase)	1.12 (1.05-1.20)	0.001	1.12 (1.04-1.20)	0.002
IMV (present vs absent)	12.44 (3.23-47.9)	<0.001	12.62 (3.25-48.99)	<0.001

## Discussion

The novelty of our study compared to other studies is that it evaluated the time from the onset of the pandemic to the emergence of omicron in COVID-19 pregnant patients. Most patients in this study were diagnosed with COVID-19 with a positive PCR test, and a small proportion with symptoms and typical radiological findings of COVID-19. After November 2021, the omicron variant began to be widely seen in Turkey. In the study in which COVID-19 pregnant and nonpregnant critically ill patients were included in the literature, the mean age and gestational age of the pregnant women were similar to the results of our study [7]. There was no difference between the periods in the mean age and mean gestational age of the patients because, as in other studies, all patients in this study were of fertility age. There was no difference between the periods in the number of pregnant women admitted to the ICU in the first two trimesters and in the third trimester. Furthermore, the rate of pregnant women in the third trimester was higher than the pregnant women in the first two trimesters in all three periods. In previous studies, hypoxemia and oxygen requirement were higher in third-trimester pregnant patients who required hospitalization and ICU [8,9]. In a review on pregnant with COVID-19, it was emphasized that pregnant women in the third trimester are the most sensitive period for COVID-19 infection [10], as third-trimester pregnant women are more prone to COVID-19 and may have the disease more severely. Considering that some physiological changes that occur during pregnancy, such as diaphragm elevation, decreased functional residual volume, relaxation of ligaments in the ribs as in all ligaments, increased pulmonary hypertension, increase the susceptibility to respiratory tract infections, this tendency increases as the pregnancy progresses [11]. In addition, changes in cellular immunity also suppress the immunity of pregnant women [12]. Therefore, it is expected that patients will present to the hospital with more serious symptoms in the last trimester and the need for intensive care will arise.

COVID-19 can present with a variety of symptoms such as cough, fever, headache, rhinitis, sore throat, and myalgia, and symptoms of the disease were reported in pregnant women, respectively [7]. In another study, fever, weakness, lack of smell and taste, and sore throat were found [13]. In the present study, unlike other studies, the most common symptom was determined as dyspnea, as the population consisted of only patients needing intensive care and the reason for admission to the ICU was respiratory failure or hypoxemia. In addition, patients need intensive care in the late period, when the respiratory decompensation occurs, rather than the early period when the virus enters the body [14]. Dyspnea was followed by cough and fever, and patients presented with similar symptoms in all periods. Although the virus mutates over time, it still continues to show similar symptoms.

In previous studies, obesity was found as the most common comorbidity [7,15]. In this study, we could not evaluate the body mass index (BMI) of the patients because we could not access the height and weight data records. Therefore, we found hypothyroidism to be the most common comorbidity we evaluated. The number of patients with gestational diabetes and hypertension from other comorbidities was also low. This may be due to insufficient records.

The IMV requirement rate of the patients was 44.95% in this presented study. This rate was similar to the results reported by Jering et al. in their study. Also, in a comprehensive study comparing pregnant patients with and without COVID-19, they reported that 212 of 6,380 COVID-19 pregnant patients needed intensive care. They found that the IMV was required in 40.56% (n=86) of 212 COVID-19 pregnant patients in the ICU [9]. In a meta-analysis including pregnant patients with COVID-19, 1,373 (3.2%) patients needed intensive care and 668 (48.65%) of them required IMV. Furthermore, this meta-analysis included studies published at the beginning of the pandemic (December 1, 2019, to October 6, 2020) [5]. Our study, on the other hand, includes the periods when different variants of the virus emerged from the beginning of the pandemic to November 30, 2021. Furthermore, these findings are similar to the results of our study.

We did not find any difference between periods in terms of prone position and ECMO applied when conventional IMV strategies and treatments were insufficient in COVID-19-related acute respiratory distress syndrome (ARDS). However, in the last period when the delta variant appeared, both IMV and ECMO and prone position were applied more frequently. Nevertheless, in the last period, when the delta variant dominated, both IMV and ECMO were applied more frequently. These findings may be an indication that pregnant women are infected more frequently and the disease is more severe in this period when the delta variant becomes widespread, and that further supportive treatments are needed in the ICU.

A study from the England included pregnant patients with COVID-19 admitted to the hospital between March 1, 2020, and July 11, 2021 [16]. This period was divided into three time frames and the outcomes were compared. The first period was from March 1, 2020, to November 30, 2020, and was dominated by the wildtype variant. The second period was from December 1, 2020, to May 15, 2021, and was dominated by the alpha variant. The third and final period was from May 16, 2021, to July 11, 2021, and was dominated by the delta variant. The treatments applied to symptomatic and critically ill patients in each period and their rates were as follows, respectively: antiviral therapy in 3%, 1.9%, and 1.8%; steroid in 4.7%, 12.4%, and 14.6%; and tocilizumab in 0%, 2.3%, and 4.1% [16]. In our study, the rate of administration of antiviral agents decreased gradually in the later periods of the pandemic. Tocilizumab was not given to any patient. The usage of Anakinra did not differ between periods. Unlike this study, ours included a wider period in which the delta variant was also seen. Furthermore, no significant differences were found in the treatment protocols in the period when the delta variant was present.

COVID-19 progresses with microthrombus and thrombotic complications. Pregnancy also creates a prothombotic process. Therefore, the risk of thrombosis increases even more in pregnant women with COVID-19 in intensive care [17]. In order to prevent thrombotic complications that may develop, LMWH was given to the majority of patients in our study who had no contraindications for thromboprophylaxis treatment. During intensive care follow-up, thromboembolism was not detected in any patient.

Antenatal low-dose aspirin is recommended to reduce placenta-mediated complications in pregnancy such as preeclampsia or fetal growth restriction and preterm birth [18]. However, the Federation of Gynecology and Obstetrics (FIGO) reported that there are not enough data to recommend against the use of low-dose aspirin for placenta-mediated complications in pregnancy [19]. Despite the two different opinions mentioned above, in our results, 40.37% of the patients were given aspirin therapy. In our study, placenta-mediated complications were not observed in any COVID-19 pregnant patient who was given or not given aspirin during follow-up in the ICU.

In this study, no difference was found between the periods in terms of mortality. In the study by Jering et al., nine (4.24%) of 212 pregnant COVID-19 patients in the ICU died, and in another study published in British Medical Journal, death was reported in eight of 64 patients [9,5]. A case series in which seven of nine patients from Iran resulted in mortality is presented [20]. The Centers for Disease Control and Prevention (CDC), on the other hand, reported that there was an increase in the mortality rates of pregnant COVID-19 in the delta period (25%) compared to pre-delta period (5%) [21]. Rangchaikul and Venketaraman also declared that the delta variant may be 2.8 times more mortal [22]. While our total mortality rate was 25.6%, it was found to be 27% in the period of delta variant.

In this study, it was found that the laboratory results of the COVID-19 pregnant patients with a mortal course - profound lymphopenia, high procalcitonin, CRP, IL-6, ferritin, AST, and LDH values - were similar to the general population with a mortal course [23]. In June 2021, the CDC reported the new variant of the coronavirus, the “delta variant.” This variant was declared to be more contagious than previous variants [2]. In Turkey, where the study was conducted, the delta variant was observed at the end of June 2021. In the study by Adhikari et al., pregnant women from the delta variant were more infected than in the pre-delta period, and there was an increase in patient hospitalization [24]. In our study, in parallel with the literature data, it was found that there was a significant increase in the number of COVID-19 pregnant cases hospitalized in the ICU in the last period in which the delta variant was seen in Turkey.

In this study, the rate of pregnant patients known to have been vaccinated was quite low at all periods. This situation is not unique to the results of our study. In the study of Adhikari et al., the rate of vaccination of pregnant women was 21.4% [24]. Although this rate is higher than the rate of vaccination in our study, it is lower than the rate of vaccination of the general population.

Adhikari et al. also emphasized that the information about why the delta variant affects pregnant women so frequently is not clear and that vaccination should be increased in pregnant women [24]. Rangchaikul and Venketaraman suggest that the delta variant is more contagious in pregnant women due to reasons such as the ability of the delta variant to create more viral load in the respiratory system, pregnancy being more susceptible to COVID-19, and vaccination being less common in pregnant women compared to the general population [22].

The limitations of the study, in addition to its single-center and retrospective nature, were that only pregnant women of childbearing age were included in the study, they was not compared with non-pregnant women, and the patients' lack of vaccination information. Another limitation is that the COVID-19 variant was not known for 63% of the patients. As the virus mutated, alpha, beta, and then the delta variants became dominant in Turkey toward the end of June 2021 [2]. Variant type is not routinely checked in PCR test positive patients in Turkey. Variant type is determined randomly. In addition, because the study was planned retrospectively, the records of the retrospective variant type and vaccination status could not be accessed when the patient died. Because when the patient dies, access to the data on the health system is closed. If information is not recorded in the hospital database, these data cannot be accessed. Therefore, variant types and vaccination status of the patients included in the study could not be determined in all of them. Nevertheless, the delta variant was found to be the most frequently detected variant in this study. This variant emerged in the last period of the study.

## Conclusions

Pregnant women became infected with COVID-19 more frequently and intensive care was needed more during the period when the delta variant was dominant in the pandemic. Mechanical ventilator requirement was also determined as a factor affecting mortality in these patients with ARDS. The continuation of comprehensive studies in the future due to ongoing mutations is very important in terms of determining the approach strategy for pregnant women with COVID-19.
